# Acute motor and sensory axonal neuropathy in association with primary Sjögren’s syndrome: a case report

**DOI:** 10.1186/s12883-021-02190-z

**Published:** 2021-04-15

**Authors:** Yu-Ming Chen, Kuei-Ying Su

**Affiliations:** 1 Department of Neurology, Hualien Tzu Chi Hospital, Buddhist Tzu Chi Medical Foundation, Hualien, Taiwan; 2 School of Medicine, Tzu Chi University, Hualien, Taiwan; 3 Division of Allergy, Immunology & Rheumatology, Hualien Tzu Chi Hospital, Buddhist Tzu Chi Medical Foundation, Hualien, Taiwan

**Keywords:** Acute motor and sensory axonal neuropathy, Guillain-Barré syndrome, Sjögren’s syndrome

## Abstract

**Background:**

Primary Sjögren’s syndrome is a chronic, autoimmune, connective tissue disorder that results from the infiltration of exocrine glands, especially the lacrimal and salivary glands, by autoantibodies. Patients with Sjögren’s syndrome commonly present with dry eyes (xerophthalmia) and dry mouth (xerostomia). However, the clinical manifestations of Sjögren’s syndrome can be complicated and variable due to involvement of extraglandular organ systems, such as the nervous system. The neurological manifestations of this disorder often precede those of other exocrine gland symptoms. Hence, early diagnosis of Sjögren’s syndrome remains a challenge.

**Case presentation:**

We report the case of a 63-year-old woman with primary Sjögren’s syndrome who presented with acute motor and sensory axonal neuropathy (AMSAN). Treatment with glucocorticoids and immunosuppressants partially improved her muscle weakness and paresthesia.

**Conclusions:**

This case demonstrates the importance of early recognition and diagnosis of AMSAN in association with primary Sjögren’s syndrome to achieve a favorable clinical outcome. Primary Sjögren’s syndrome may be underdiagnosed because of vague symptoms of the sicca complex. Comprehensive immunological testing to evaluate this condition may be performed in patients presenting with variants of Guillain-Barré syndrome.

## Background

Primary Sjögren’s syndrome (pSS) is a chronic autoimmune disease characterized by the presence of autoantibodies to Sjögren’s syndrome A (SSA) and SSB antigens with systemic manifestations reflecting the organs affected. Patients with pSS typically present with dysfunction of the exocrine glands, predominantly the lacrimal and salivary glands, in the form of dryness of the eyes (xerophthalmia) and mouth (xerostomia). However, this disease can also affect non-exocrine organ systems and cause various clinical manifestations. It can also affect the central and peripheral nervous systems [1–3]. Neurological manifestations often precede exocrine gland symptoms such as xerophthalmia and xerostomia [4–6].

Here, we report the case of a 63-year-old woman who presented with fulminant and acute motor and sensory axonal neuropathy (AMSAN) and was subsequently diagnosed with pSS. She received immunosuppressive therapy and achieved partial improvement of the neurological deficits.

## Case presentation

The patient was a 63-year-old woman with a history of adenomyosis, gout, and essential hypertension. She could previously perform basic activities of daily living (BADL) independently. Insidiously, she became symptomatic with dry eyes (keratoconjunctivitis sicca). She initially presented with “pins- and- needles” sensation (paresthesia) over the bilateral palms and feet, which developed simultaneously. These symptoms were followed by progressive weakness in the bilateral arms and legs distally more than proximally. This severely impaired her BADL and caused her to be wheelchair-bound.

After 10 days of progressive weakness in all four limbs, she exhibited poor muscle strength, which rendered her unable to stand and walk. She became completely wheelchair-dependent, which compelled her to visit our neurology clinic. The neurological examination revealed acute progressive weakness/paresthesia in all the four limbs, with generalized hyporeflexia. Nerve conduction study (NCS) of her bilateral upper and lower limbs revealed axonal sensorimotor polyneuropathy and severe bilateral distal median neuropathy at the wrists. Acute polyneuropathy was pronounced. We advised her to visit our emergency room (ER) for further examination, but she refused further clinical evaluation due to personal reasons. Her medical history indicated that she had been admitted to our Ophthalmology department from July 17–27, 2020, for the following reasons 1) corneal ulcer with corneal perforation in the right eye status after corneal suture and tissue glue, 2) purulent endophthalmitis in the right eye following intravitreal injection of antibiotics, and 3) superficial punctate keratitis (SPK) in the left eye.

**Fig. 1:**
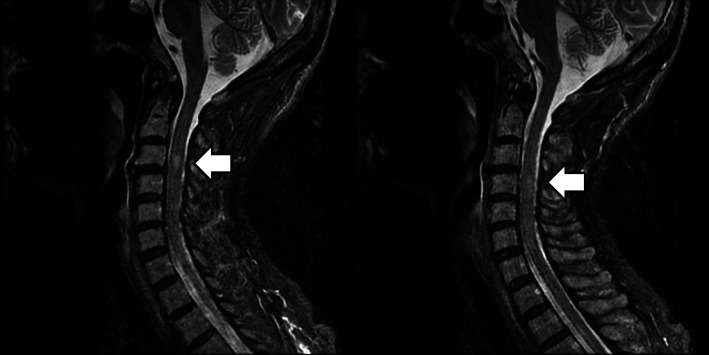
T2 weighted cervical magnetic resonance imaging (MRI) without contrast. Cervical spine MRI showing mild increase in the T2-signal intensity of the cervical cord at the C3–C4 levels

On August 4, 2020, she was sent to our ER due to acute urinary retention and progressive weakness in all limbs. In the ER, the neurological examination revealed clear consciousness, incomplete closure of the left eyelid, no bulbar signs and symptoms, and quadriplegia with muscle strength scores as follows: upper limbs proximal-2, distal-3 and lower limbs proximal-1, distal-2, as per the Medical Research Council Scale (MRC) scale, generalized absence of deep tendon reflexes, and paresthesia/impaired joint position sense with glove-and-stocking distribution. Cervical and thoracic spine magnetic resonance imaging (MRI) revealed old compression fractures at T6–T7 and mildly hyperintense T2 lesion at the C3–C4 level (Fig. 1).

Based on the initial clinical presentation, Guillain-Barré syndrome (GBS) was suspected. Estimation of the levels of serum tumor markers, autoimmune profile tests, NCS of the bilateral upper and lower limbs, stool culture, orbital MRI with contrast, and lumbar puncture were performed to establish accurate diagnosis and exclude other possibilities, such as transverse myelitis and central nervous system infection.

Serum tumor markers and autoimmune profiles yielded high titers of anti-SSA and anti-SSB autoantibodies (Table 1). NCS of the bilateral upper and lower limbs revealed severe axonal sensorimotor polyneuropathy and severe bilateral distal median neuropathy at the wrists, with a lower amplitude than that in the previous study. Orbital MRI results were negative. Stool culture was negative for *Campylobacter*. Lumbar puncture consistently revealed albuminocytologic dissociation, compatible with suspected GBS. Based on the clinical manifestations, electrodiagnostic criteria [7], and cerebrospinal fluid analysis, we tentatively diagnosed the patient with AMSAN-variant GBS.

**Table 1:** Autoimmune serological test results

Antibody	Values	Normal values
Anti-nuclear antibody	1:640 (+), homogenous	< 1:40
Anti-SSA (EliAU/mL)	> 240	10
Anti-SSB (EliAU/mL)	107	10
Anti-CENP (EliAU/mL)	0.6	10
Anti-DNA (IU/mL)	0.8	15
Anti-RF (IU/mL)	< 10	14
Anti-TPO (IU/mL)	0.6	9
Anti-TG (IU/mL)	< 0.9	4
Lupus anticoagulant	1.2	1.2
Anti-RNP (EliAU/mL)	1.2	10
Anti-Smith (EliAU/mL)	0.9	10
c-ANCA (IU/mL)	0.2	3
p-ANCA (IU/mL)	0.2	5
Anti-Cardiolipin-IgG (GPL U/mL)	< 1.6	20
Anti-Cardiolipin-IgM (MPL U/mL)	7.1	12.5
Anti-β2GP1-IgG (U/mL)	< 1.4	20
Anti-β2GP1-IgM (U/mL)	1.2	20
Anti-AQP4	Negative	Negative

Serologic tests revealed positive anti-SS-A and anti-SS-B antibodies

*AQP4* aquaporin 4, *β2GP1-IgG* beta2 glycoprotein IgG, *β2GP1-IgM* beta2 glycoprotein IgM, *CENP* Centromere IgM, *c-ANCA* cytosolic anti-neutrophil cytoplasmic antibody, *Cardio-IgG* Cardiolipin IgG, *Cardio-IgM* Cardiolipin IgM, *p-ANCA* perinuclear anti-neutrophil cytoplasmic antibody, *RF* Rheumatoid factor, *RNP* Ribonuclear protein, *SSA* Sjögren’s syndrome A, *SSB* Sjögren’s syndrome B, *TPO* Thyroid peroxidase, *TG* Thyroglobulin

The patient did not consent to plasma exchange. Therefore, intravenous immunoglobulin (IVIG) treatment was considered. Meanwhile, intravenous methylprednisolone (1000 mg/day) was prescribed on the third day of admission. The steroid pulse therapy lasted for 5 days, during which the left upper distal limb muscle strength score improved to MRC scale 3. Bilateral feet and right palm paresthesia ameliorated, and the right leg muscle strength score improved to MRC scale 3 over the ensuing days. A rheumatologist was consulted regarding the elevated serum levels of anti-SSA and anti-SSB autoantibodies. According to the 2016 ACR/EULAR classification criteria for pSS [8], the patient had high titers of anti-SSA antibodies as well as corneal ulcers with severe SPK in the left eye as a surrogate of ocular staining score or van Bijsterveld score. Thus, the rheumatologist was able to diagnose pSS and also classify it.

We administered IVIG after steroid pulse therapy. There was a remarkable improvement in the proximal limb muscle strength on the third day of IVIG administration. Moreover, the patient’s clinical status significantly improved over the next few days. Continuous improvement in muscle power in all four limbs proximally as well as distally was noted. The patient also regained the use of axial muscles. Therefore, the rehabilitation program was upgraded due to her progress; however, she refused further transfer to the rehabilitation department as she wanted to go home early. Finally, the patient was discharged after 37 days of admission with partially-dependent BADL.

During the follow-up at the neurology outpatient clinic 2 weeks after discharge, the patient’s muscle strength progressively improved (upper proximal-3, distal-3, lower proximal-4, distal-3), and the incomplete left eye closure resolved.

## Discussion and conclusions

pSS is a chronic, autoimmune disease that usually occurs in middle-aged adults (mean age of onset: 4th to 5th decade of life) and is predominant in females. Its clinical picture varies among affected individuals. The most common symptoms are xerophthalmia and xerostomia. According to the 2016 ACR/EULAR classification criteria [8], any individual who fulfills the inclusion criteria with a weighted summed score of ≥4 can be diagnosed with pSS.

Most patients with pSS initially experience neurological symptoms, wherein both peripheral and central nervous systems could be affected [1–3]. These symptoms vary widely, including axonal sensory neuropathy, axonal sensorimotor neuropathy, GBS, small- fiber neuropathy, and demyelination [5, 6]. If the sicca symptoms (xerophthalmia and xerostomia) are subclinical and vague, diagnosis of pSS may be delayed or underdiagnosed.

There are multiple hypotheses regarding the neurological symptoms associated with the pathogenicity of pSS [6], including small-vessel vasculopathy [9], dorsal root ganglionitis [10], cryoglobulinemia [11], demyelination [12], myelitis [13], and antibody-mediated autonomic dysfunction [14]. This heterogeneity of the underlying neuropathological mechanisms of pSS complicates the treatment approach.

AMSAN is a rare form of GBS variants. The pathology is predominantly axonal loss of both motor and sensory nerve fibers. Although AMSAN has characteristics similar to those of acute motor axonal neuropathy (AMAN), the onset of AMSAN is rapid and its symptoms are more severe, resulting in significant disability [15]. AMSAN and AMAN are associated with *Campylobacter jejuni* infection, which in itself is a poor prognostic factor [16].

It is possible that our patient coincidentally had both AMSAN and pSS, although there may be an association between them. After we excluded other causes of acute polyneuropathy, we concluded that AMSAN may be associated with pSS based on the autoimmune serologic data and ocular lesions. Notably, the subclinical cervical myelitis observed on cervical MRI may be regarded as an accidental finding of several neurological conditions associated with Sjögren’s syndrome [1–3].

As mentioned in an earlier study, the first line of treatment for GBS is immunotherapy with immunoglobulin or plasma exchange, with no significant differences between the two with regard to the clinical outcomes [17]. Currently, there is no known evidence of the beneficial effects of corticosteroids in the treatment of this condition [18, 19]. A meta-analysis of six trials comprising 587 participants, including patients with GBS, revealed no significant improvement in the disability score of patients treated with glucocorticoids compared with those who were not treated with these drugs [20]. However, the best treatment for AMSAN is unknown as there are very few studies available on this topic in the literature.

Methylprednisolone pulse therapy was prescribed for our patient after she refused plasma exchange. Mild improvement with no worsening of weakness and paresthesia in the limbs was noted. Subsequently, we administered IVIG for definite immunosuppression. The clinical response of the patient to this treatment was excellent, and she demonstrated good antigravity muscle strength in her limbs and residual mild right palmar paresthesia upon discharge. Although the early use of immunosuppressive therapy helped achieve clinical improvement during the acute stage, it did not completely prevent AMSAN-related functional damage. However, during the follow-up, the AMSAN sequelae continued to improve.

In this report, we presented a case of AMSAN with pSS and demonstrated that limb weakness may be improved by immunosuppressive therapy. The notable points in our case report are as follows: (i) the neurological manifestations of pSS precede the sicca symptoms; (ii) the symptoms vary widely, from AMSAN to sub-acute myelitis; and (iii) early and aggressive immunosuppressive therapy resulted in a remarkable clinical response in the patient.

This case emphasizes the importance of comprehensive immunological testing for patients who present with variants of GBS.

## Data Availability

All data generated or analyzed during this study are included in this article.

## Abbreviations

AMSAN: Acute motor and sensory axonal neuropathy
AMAN: Acute motor axonal neuropathy
BADL: Basic activities of daily living
ER: Emergency room
GBS: Guillain-Barré syndrome
IVIG: Intravenous immunoglobulin
MRC scale: Medical Research Council scale
MRI: Magnetic resonance imaging
NCS: Nerve conduction study
OD: Oculus dexter
OS: Oculus sinister
SSA: Sjögren’s syndrome-related antigen A
SSB: Sjögren’s syndrome-related antigen B
SPK: Superficial punctate keratitis
